# Supplementary material for: Structural Constraints Acting on the SARS-CoV-2 Spike Protein Reveal Limited Space for Viral Adaptation
Source: Genome Biol Evol. 2026 Mar 25;18(3):evag049. doi: 10.1093/gbe/evag049 (PMC13012802; doi:10.1093/gbe/evag049)
Supplement: evag049_Supplementary_Data [file evag049_supplementary_data.pdf]

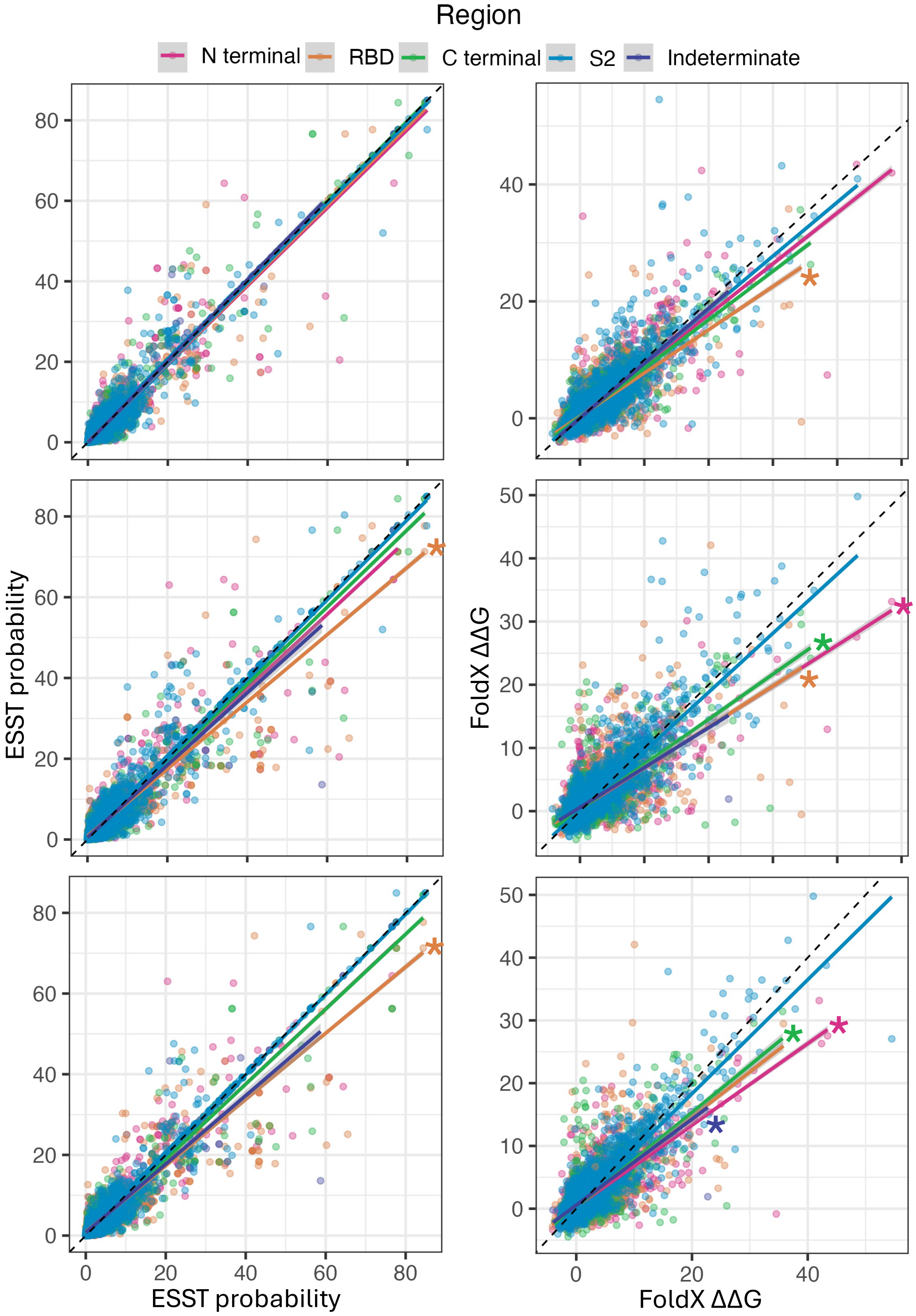

Figure S1: Scatterplots showing correlation of ESST and FoldX scores between 'up' and 'down' chains in open and closed conformation S protein structures. Scatterplots showing the correlation in ESST (left column) and FoldX (right column) scores between predictions made on open and closed conformation S proteins. The top plots show a closed conformation 'down' chain on the x-axis and an open conformation 'down' chain on the y-axis; the middle plots show a closed conformation 'down' chain on the x-axis and an open conformation 'up' chain on the y-axis; the bottom plots show an open conformation 'down' chain on the x-axis and an open conformation 'up' chain on the y- axis. Substitutions are coloured by protein region. Coloured lines show linear regressions for each protein region. Comparisons with significantly different mean values are marked with coloured asterisks. Dashed black line shows a perfect correlation.

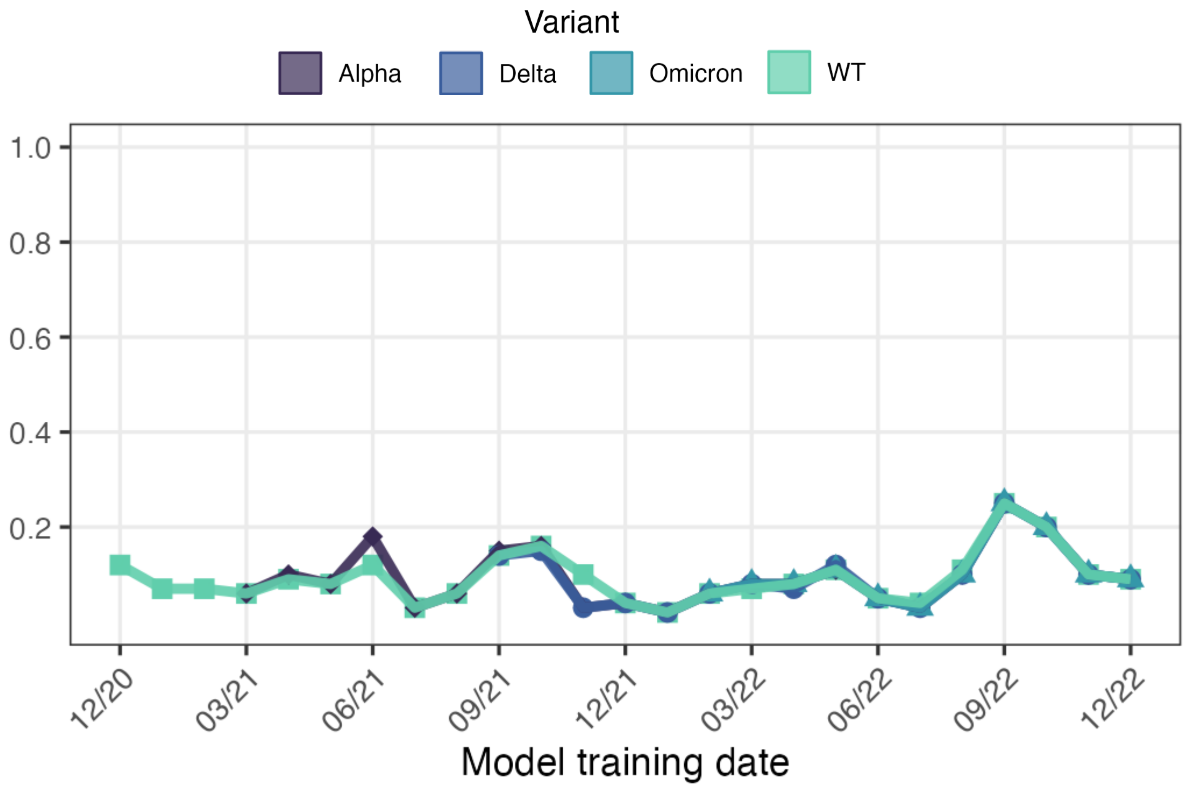

Figure S2 shows the outcomes of ML predictions of substitution class on class-switching substitutions; that is, those that change class between the train and test sets. This accounts for ~15% of substitutions. The majority of class-switching substitutions are incorrectly classified, resulting in an ROC below 0.5 when these substitutions are analysed independently.

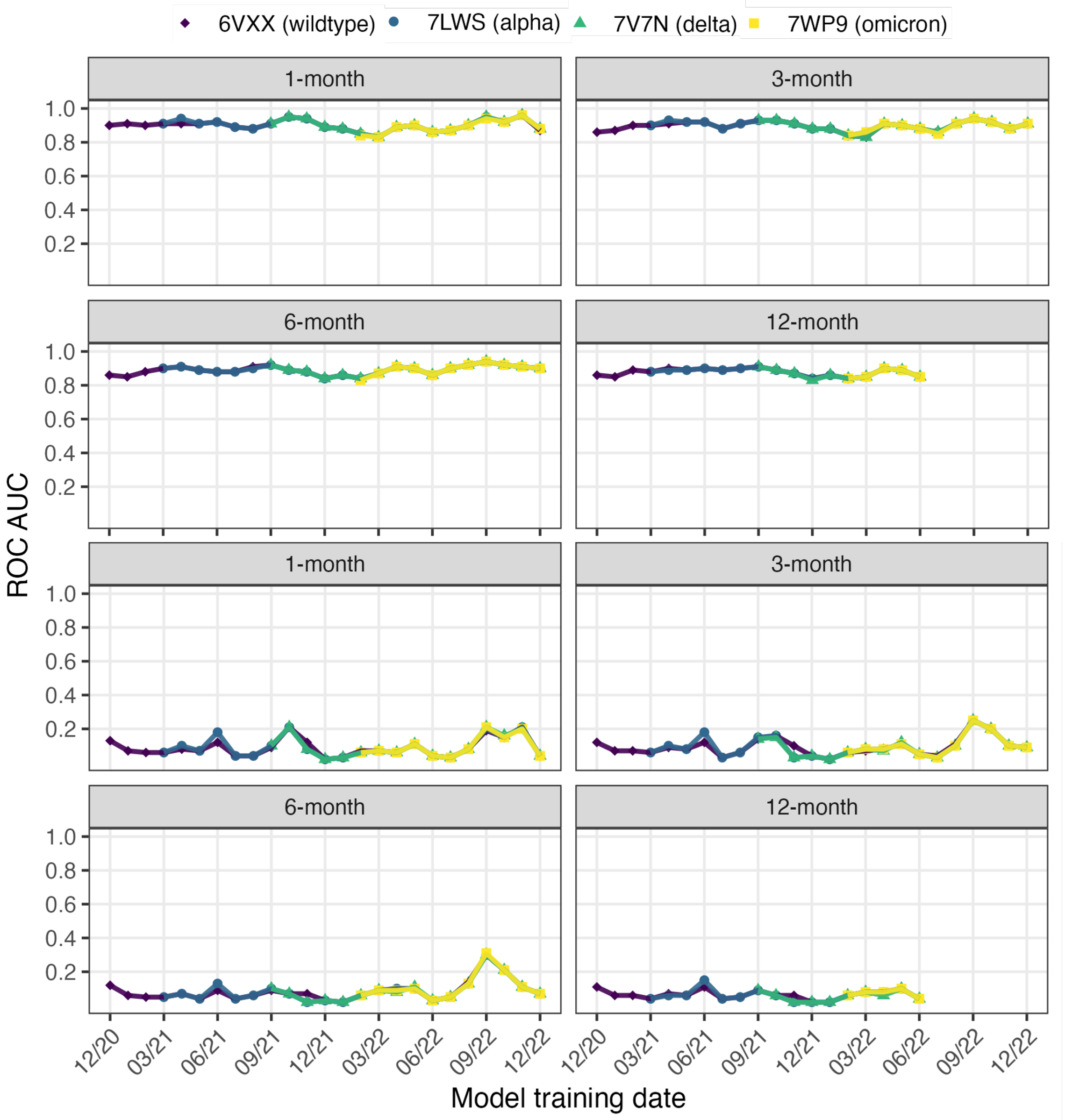

Figure S3: Results of training LOOS ensemble RF models at different cutoff dates with test sets sampled over different periods. AUC values are shown for all substiutions (**A**) and class-switching substiutions (**B**) curves calculated from ensemble models trained at different cutoff dates. Training data is generated by sampling of all sequences deposited 3-months before the cutoff date. Results are shown for model performance on four test sets generated from sequences deposited 1-, 3-, 6- and 12-months after the cutoff date. Four different input structures were used to calculate constraint predictors, these were 6VXX (WT), 7LWS (Alpha variant), 7V7N (Delta variant) and 7WP9 (Omicron variant).

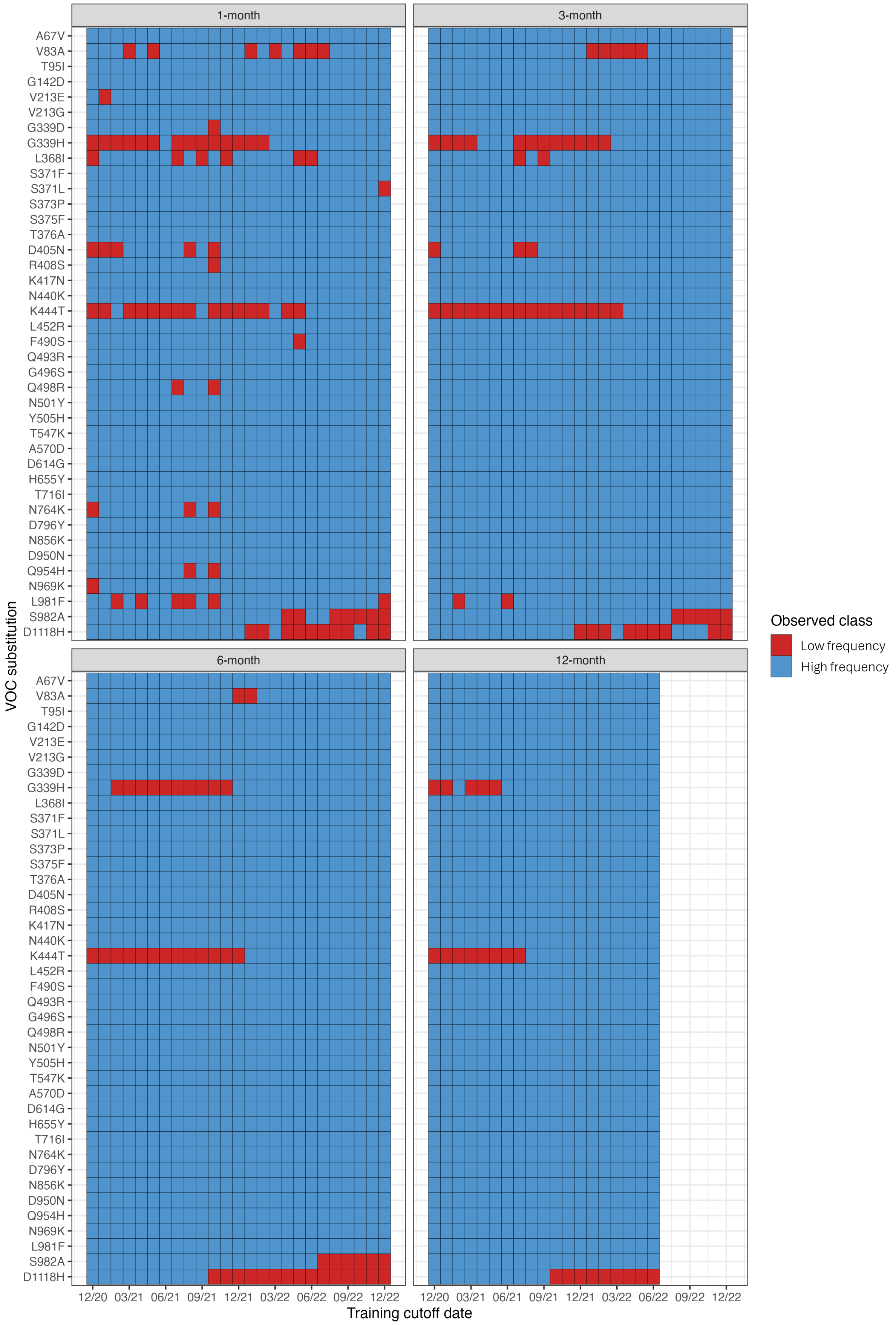

Figure S4: Classification of VOC substitutions over time. The observed class assigned to signature substitutions of VOCs are shown using the 1-month, 3-month, 6-month and 12-month test periods. Low frequency substitutions are shown as red, high frequency substitutions are shown as blue.
